# Preventing HIV infection without targeting the virus: how reducing HIV target cells at the genital tract is a new approach to HIV prevention

**DOI:** 10.1186/s12981-017-0166-7

**Published:** 2017-09-12

**Authors:** Julie Lajoie, Lucy Mwangi, Keith R. Fowke

**Affiliations:** 1 Department of Medical Microbiology and Infectious Diseases, University of Manitoba, 539-745 Bannnatyne Avenue, Winnipeg, MB R2N 1V3 Canada; 2 Department Medical Microbiology, University of Nairobi, Nairobi, Kenya; 3 Department of Community Health Science, University of Manitoba, Winnipeg, Canada

**Keywords:** HIV, Immune activation, Protection, Highly exposed seronegative (HESN), Immune quiescence

## Abstract

For over three decades, HIV infection has had a tremendous impact on the lives of individuals and public health. Microbicides and vaccines studies have shown that immune activation at the genital tract is a risk factor for HIV infection. Furthermore, lower level of immune activation, or what we call immune quiescence, has been associated with a lower risk of HIV acquisition. This unique phenotype is observed in highly-exposed seronegative individuals from different populations including female sex workers from the Pumwani cohort in Nairobi, Kenya. Here, we review the link between immune activation and susceptibility to HIV infection. We also describe a new concept in prevention where, instead of targeting the virus, we modulate the host immune system to resist HIV infection. Mimicking the immune quiescence phenotype might become a new strategy in the toolbox of biomedical methods to prevent HIV infection.

*Clinical trial registration* on clinicaltrial.gov: #NCT02079077

## Background

According to the latest UNAIDS report, 36.7 million people are living with HIV/AIDS worldwide. Despite the development of new antiretroviral drugs and better access to care and prevention programs, the number of new HIV cases has remained over 2 million per annum over the past 10 years with a very slow rate of decline [1]. Clearly, existing prevention methods are not sufficient and new approaches are required. However, to develop new biomedical prevention methods, we need a better understanding of the factors driving susceptibility to HIV infection.

## Learning from the past

### Immune activation and susceptibility to HIV infection

Mucosal surfaces of the genital and gastrointestinal tracts are the major routes of entry of HIV. In general, the presence of a pathogen leads to recruitment and activation of immune cells at the site of infection leading to the elimination of the pathogen. In the case of HIV infection, this recruitment and cell activation serves to increase the number of HIV target cells thereby actually facilitating the establishment of an infection. As such, increased immune activation is considered an important risk factor for acquiring HIV [2].

The presence of sexually transmitted infections (STIs) is associated with susceptibility to HIV infection [3, 4]. This increased risk is due to the presence of micro-lesions caused by the pathogen, which may facilitate HIV entry, or by the recruitment of activated immune cells to the site of infection, which increases the pool of HIV target cells [5]. For instance, infection by *Neisseria gonorrhoea* elicits a Th17 response [6] that is associated with an influx of neutrophils and a pro-inflammatory milieu [7]. This response aims to destroy *Neisseria gonorrhoea* infection. However, as Th17 T cells are highly susceptible to HIV infection, this fight against gonorrhoea increases the susceptibility to HIV infection [8]. Likewise, bacterial vaginosis (BV) increases the risk of acquiring HIV by 60%. BV increases the expression of IL-1α, IL-1β and TNF-α at the genital mucosa [9] which helps HIV replication. Herpes simplex virus-2 is associated with a significant increase in the frequency of mucosal HIV target cells (CD4+ CCR5+ T cells) [10]. Overall, studies have demonstrated that the immune fight against STIs modifies the genital milieu toward an inflammatory environment, which increases susceptibility to HIV.

Over the last 20 years, different microbicides have been tested to prevent HIV infection. However, most microbicide studies failed to prevent HIV infection and worse, some increased the risk of infection. The best known failed microbicide was the nonoxynol-9 (N-9). The gel did not reduce the rate of STIs rather showed that N-9 increases genital lesions and the risk of gonorrhoeal and HIV infection [11]. Later, it was shown that N-9 causes cervical epithelium damage [12] and increases the expression of pro-inflammatory cytokines/chemokines such as MCP-1, IL-8, RANTES, IL-12, L-selectin and VCAM [13]; as well as promoting HIV transmission through interleukin mediated NF-ϰB activation [14].

More recently, the CAPRISA 004 clinical trial analysed the efficacy of a 1% tenofovir gel formulation used before and after sexual intercourse. Overall, the study showed a 39% reduction in HIV infections and a 54% reduction was observed among the women who showed high adherence to the study protocol [15]. Interestingly, it was also observed that, independent of the study arm, higher risk of sero-conversion was associated with pre-existing mucosal immune activation [16]. Masson et al. showed that later sero-conversion was associated with increased expression of IL-8, MIP-1α, MIP-1β and interferon γ inducible protein (IP)-10 in the vaginal milieu [17] in samples obtained prior to HIV infection, and concluded that an existing inflammatory environment was associated with an increased risk of HIV acquisition.

**Fig. 1:**
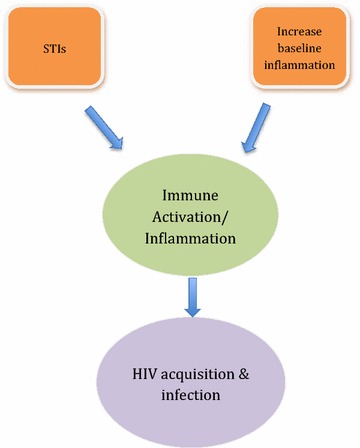
A model of the relationship between immune activation/inflammation and HIV-1 infection. Immune activation and a pro-inflammatory state drive HIV-1 acquisition and infection

Further evidence that pre-existing immune activation is a risk factor for HIV infection comes from the vaccine field. The STEP trial tested the efficacy of a DNA-based prime boost vaccine—MRK AD5^®^ HIV-1 *Gag/Pol/Nef*. The study enrolled 3000 HIV-negative participants at high risk of HIV infection. Results showed that, after vaccination, risk of infection was associated with prior Ad5 sero-positivity and lack of circumcision. Detailed immune analyses showed that seroconversion was associated with elevated levels of non-specific IFNγ stimulation (i.e. immune activation) [18, 19]. Together the N-9, CAPRISA 004 microbicide and STEP vaccine trial show that immune activation is strongly associated with increased susceptibility to HIV infection (Fig. 1). This relationship must be taken into account to develop an efficient preventative vaccine/microbicide.

#### Preventing inflammation is protective

In the last section, we highlighted some studies that demonstrated that inflammation is associated with an increased the risk of HIV infection. However, what is the evidence that preventing inflammation is protective? Are there human examples that support a low immune activation environment is more resistant to HIV infection?

### Learning from HIV exposed sero-negative (HESN) individuals

Despite being at high risk of infection, some individuals remain HIV uninfected. Known as HIV-exposed sero-negatives (HESN), groups of female sex workers (FSWs), HIV discordant couples, children born to HIV infected mothers and men who have sex with men (MSM) exhibit natural protection against HIV infection [2, 20–22]. One such HESN group is a group of FSWs from the Pumwani cohort in Nairobi, Kenya who have been extensively studied over the last 30 years. We demonstrated that HIV uninfected women enrolled in this cohort for a period of 7 years or more had a reduced risk of acquiring HIV [23]. Recently, McKinnon et al. showed that while HIV prevalence in Nairobi has drastically decreased, statistical modelling analysis indicates 23% reduction in risk of sero-conversion for each year of sex work [24]. Those women represent an extreme phenotype of HESN.

**Fig. 2:**
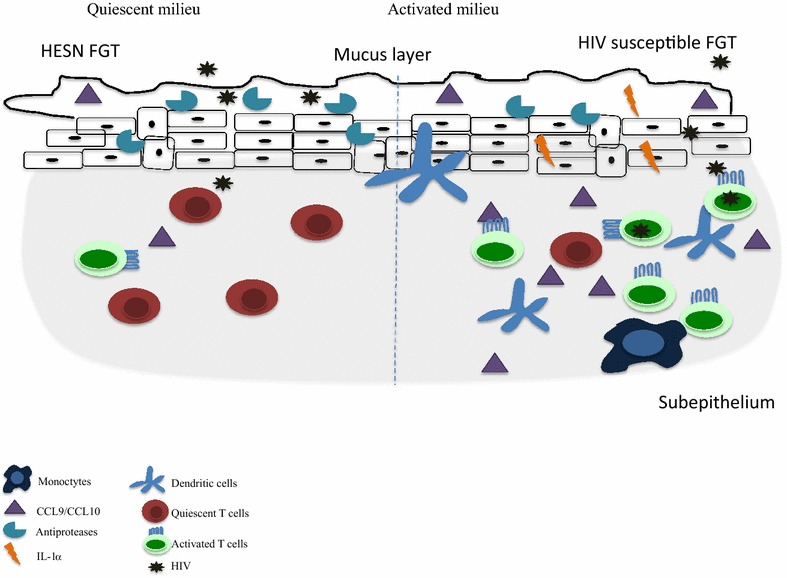
Schematic representation of the immune quiescence phenotype observed at the mucosal compartment in HESN from the Pumwani sex worker cohort

HESNs in this Nairobi cohort have reduced gene expression in the T cell receptor-signalling pathway, which is crucial to T cell activation [25, 26]. Furthermore, non-stimulated peripheral blood mononuclear cells isolated from HESNs expressed lower levels of IL-1β, IL-6 and TNF cytokines compared to susceptible individuals [25]. Card et al. showed HESNs displayed significantly lower proportion of activated T cells (CD4+ CD69+ and CD8+ CD69+) and higher levels of T regulatory cells (CD4+ CD25+ FOXP3+) when compared to the HIV-negative controls [27]. At the genital tract, HESNs have lower levels of inflammatory chemokines such as CCL9, IL-1α, and CCL10 [28] and higher levels of innate anti-inflammatory antiproteases [29]. This unique phenotype of lower baseline T cell activation was named immune quiescence (IQ) (Fig. 2).

This IQ phenotype has also been observed among HESNs from other cohorts. In the Amsterdam Cohort Studies, it was shown that HESNs had lower proportions of systemic CD4+ CCR5+ T cells. [30]. HESNs from Côte d’Ivoire had lower expression of CD69+ on memory T cells and significantly lower expression of pro-inflammatory cytokines [31]. More recently, it was shown that HESNs from a cohort of FSWs in Benin had higher levels of mucosal tolerogenic myeloid cells, higher levels of regulatory T cells [32] and reduced mucosal levels of TNF-α and IFN-γ in HESNs [33].

Overall, these findings suggest that natural protection against HIV infection is associated with a lower immune activation state. It is important to mention that IQ correlates with a lower baseline of T cells activation, but not with immunosuppression. Indeed, the Amsterdam Cohort Study indicated that this low state of T cell activation does not hinder the ability of the immune system to respond to pathogens [30].

So far we have provided evidence that genital inflammation is a risk factor for HIV infection, and that models of natural protection display a quiescent immune environment. Is it possible to induce this IQ phenotype?

### The future of HIV prevention: inducing immune quiescence?

In order to reduce levels of immune activation, we explored the possibility of using safe, affordable, non-stigmatizing and globally accessible anti-inflammatory drugs to induce an immune quiescent phenotype similar to the one observed in HESN. To address this we conducted a pilot study to investigate the ability of low doses of daily-administered hydroxychloroquine (HCQ) (200 mg/day) or acetylsalicylic acid (ASA) (81 mg/day) to induce this T cell IQ phenotype systemically and at the mucosal level (Register #NCT02079077, ethics approved by Universities of Manitoba and Nairobi). Preliminary analysis indicates that there was a reduction in the proportion of HIV target cells at the genital tract similar to levels observed in the HESN cohort (eposter #P06.05 and P19.25 presented at HIV R4P 2016, Chicago, USA; unpublished data). Further studies are required to determine the mechanism of the effect of ASA, including on innate immune cells, and to assess if that level of HIV target cell reduction is indeed protective. Importantly, our study does provide evidence that it is possible to reduce the level of HIV targets cells at the genital tract using anti-inflammatory drugs.

Reducing HIV target cells at the genital tract is a new concept in HIV prevention. It uses safe and globally accessible drugs that are not associated with HIV prevention and, therefore, are not stigmatizing, which is a problem encountered with the current pre-exposure prophylaxis (PrEP) using anti-retroviral drugs. Another advantage is that this approach does not target the virus; therefore, viral mutation is unlikely to provide escape variants. Care must be taken to ensure that reducing inflammation does not increase the risk of acquiring other infections. However, to date there is no evidence that the hundreds of thousands of people taking HCQ and ASA for long-term prevention of inflammatory and cardiovascular conditions, respectively, are more susceptible to infections.

While reducing inflammation to decrease HIV target cells in the genital tract would not be the primary HIV prevention approach for all at-risk individuals, it could provide an additional tactic among some individuals that could be used alone or in conjunction with other behavioural and biomedical prevention approaches such as microbicides or vaccines. Providing at-risk individuals, especially women, with a wider selection of safe and effective HIV prevention tools that they control, and are comfortable with, is a goal we must reach if we are to significantly decrease the HIV incidence rates that have stagnated over the last decade of the HIV pandemic.

## Abbreviations

HIV: human immunodeficiency virus
HESN: highly-exposed seronegative
AIDS: acquired immuno-deficiency syndrome
BV: bacterial vaginosis
N-9: nonoxynol-9
STI: sexually transmitted infections
CS: cellulose sulphate
IP: interferon γ inducible protein
GML: glycerol monolaurate
SIV: simian immunodeficiency virus
DNA: deoxyribonucleic acid
FSW: female sex worker
IQ: immune quiescence
IRF-1: interferon regulatory factor-1
PrEP: pre-exposure prophylaxis
CIHR: Canadian Institute of Health Research
